# Early Detection of Microvascular Changes in Patients with Diabetes Mellitus without and with Diabetic Retinopathy: Comparison between Different Swept-Source OCT-A Instruments

**DOI:** 10.1155/2019/2547216

**Published:** 2019-06-12

**Authors:** Stela Vujosevic, Caterina Toma, Edoardo Villani, Valentina Gatti, Marco Brambilla, Andrea Muraca, Maria Chantal Ponziani, Gianluca Aimaretti, Alessandro Nuzzo, Paolo Nucci, Stefano De Cilla'

**Affiliations:** ^1^Eye Clinic, University Hospital “Maggiore della Carità”, Novara, Italy; ^2^Department of Clinical Sciences and Community Health, University of Milan, Milan, Italy; ^3^Eye Clinic, San Giuseppe Hospital, Milan, Italy; ^4^Department of Medical Physics, University Hospital “Maggiore della Carità”, Novara, Italy; ^5^Diabetology Unit, S.S. Trinità Hospital, Borgomanero, Italy; ^6^Department of Translational Medicine, Endocrinology, University Hospital “Maggiore della Carità”, Novara, Italy; ^7^Department of Health Sciences, University of East Piedmont “A. Avogadro”, Novara, Italy

## Abstract

Optical coherence tomography angiography (OCT-A) has recently improved the ability to detect subclinical and early clinically visible microvascular changes occurring in patients with diabetes mellitus (DM). The aim of the present study is to evaluate and compare early quantitative changes of macular perfusion parameters in patients with DM without DR and with mild nonproliferative DR (NPDR) evaluated by two different swept-source (SS) OCT-A instruments using two scan protocols (3 × 3 mm and 6 × 6 mm). One hundred eleven subjects/eyes were prospectively evaluated: 18 healthy controls (control group), 73 eyes with DM but no DR (no-DR group), and 20 eyes with mild NPDR (DR group). All quantitative analyses were performed using ImageJ and included vessel and perfusion density, area and circularity index of the FAZ, and vascular complexity parameters. The agreement between methods was assessed according to the method of Bland-Altman. A significant decrease in the majority of the considered parameters was found in the DR group versus the controls with both instruments. The results of Bland-Altman analysis showed the presence of a systemic bias between the two instruments with PLEX Elite providing higher values for the majority of the tested parameters when considering 6 × 6 mm angiocubes and a less definite difference in 3 × 3 mm angiocubes. In conclusion, this study documents early microvascular changes occurring in the macular region of patients at initial stages of DR, confirmed with both SS OCT-A instruments. The fact that early microvascular alterations could not be detected with one instrument does not necessarily mean that these alterations are not actually present, but this could be an intrinsic limitation of the device itself. Further, larger longitudinal studies are needed to better understand microvascular damage at very early stages of diabetic retinal disease and to define the strengths and weaknesses of different OCT-A devices.

## 1. Introduction

Diabetic retinopathy (DR) is the most important complication of both type 1 and type 2 diabetes mellitus (DM) [1]. Currently, there is a growing body of scientific evidence indicating that specific neural and vascular retinal modifications can be present even before the onset of clinically visible signs of DR [2–11].

Recent advent of optical coherence tomography angiography (OCT-A), a new noninvasive depth-resolved retinal imaging technique, has allowed a better evaluation of the changes occurring at the macular and peripapillary capillary networks in patients with DM with or without DR [12–17]. Subclinical and early microvascular changes detected on OCT-A mainly consist of remodeling and enlargement of the foveal avascular zone (FAZ), capillary nonperfusion, and reduced vascular density [10, 12–21], and recently, also venous beading and increased vascular tortuosity were found to be more frequent in the macular region of patients with DM but with no DR versus healthy controls [10].

Several studies were performed using different OCT-A devices, and this could explain some discrepancies in the available results. In fact, even if all OCT-A devices rely on the common principle that erythrocytes could be used as a motion contrast to differentiate vessels from static tissues [22], they use different algorithms for image acquisition and processing and different methods for layer segmentation [23–27]. Recently, Corvi et al. evaluated the reproducibility of quantitative parameters using seven different OCT-A devices in healthy subjects and concluded that the measurements obtained were too different to allow reliable comparisons [28].

The aim of this study is to evaluate and compare early quantitative changes of the macular perfusion parameters in patients with DM without DR and with mild nonproliferative DR (NPDR) by two different swept-source (SS) OCT-A instruments and using two scan protocols (3 × 3 mm and 6 × 6 mm).

## 2. Materials and Methods

### 2.1. Patients and Study Design

In this prospective cross-sectional comparative case-control study, we consecutively enrolled 111 eyes of 111 subjects, consisting of 18 healthy control eyes (control group), 73 eyes with DM without clinical signs of DR (no-DR group), and 20 eyes with mild NPDR (DR group). The right eye was considered for the analysis, unless a better quality in the left eye images was present. All patients with DM were referred from the Diabetes Unit to the Medical Retina Service, University Hospital “Maggiore della Carità,” Novara, Italy, for evaluation. Normal controls were recruited among subjects referring to our clinic for a routine annual examination or for preliminary exams for cataract surgery (the eye that was not planned for surgery was chosen for the study).

Inclusion criteria for the study were as follows: patients over 18 years of age with a diagnosis of type 1 or type 2 DM according to the updated diagnostic criteria by the American Diabetes Association [29] and confirmed by an expert diabetologist (G.A., M.C.P., and A.N.); no signs of DR or signs of mild NPDR on slit-lamp fundus examination with 90D lens (Volk Optical Inc., Mentor, OH, USA) performed by an expert ophthalmologist (S.V.) according to the *International Clinical Diabetic Retinopathy Disease Severity Scale* [30]; and subjects with normal glucose test for the control group. Exclusion criteria were as follows: any retinal disease other than mild NPDR (including diabetic macular edema); any previous intraocular treatment (such as intravitreal injections of anti-VEGF/steroids or retinal laser); cataract surgery within 6 months in the study eye; refractive error of greater than +/−4D; glaucoma or history of ocular hypertension (IOP > 21 mmHg); neurodegenerative diseases (e.g., multiple sclerosis, Alzheimer's disease, and Parkinson's disease); uncontrolled systemic blood pressure (BP ≥ 120/80 mmHg) [31]; and poor quality of OCT and/or OCT-A images due to significant media opacity or poor patient cooperation.

Anamnestic data were collected for each patient, including type of DM, value of glycated haemoglobin (HbA1c), use of antidiabetic agents (insulin and/or oral hypoglicaemic drugs), use of other drugs for concomitant pathologic conditions (e.g., systemic hypertension, cardiovascular diseases, and rheumatic diseases), and previous ocular or other surgery. Each patient underwent a complete ophthalmologic examination including best-corrected visual acuity (BCVA) determination using the standard Early Treatment Diabetic Retinopathy Study (ETDRS) protocol at 4 meters distance, IOP measurement, slit-lamp dilated fundus examination with 90D lens, and acquisition of color fundus photography of the posterior pole. On the same day, SS-OCT and SS-OCT-A images were acquired with two different instruments.

The study adhered to the tenets of the Declaration of Helsinki and was approved by the institutional Ethics Committee (CE123 2017); each patient approved to participate in the study and signed a written informed consent.

### 2.2. Imaging

#### 2.2.1. Swept-Source Optical Coherence Tomography and Optical Coherence Tomography Angiography

On the same day, each patient underwent OCT and OCT-A with two different SS instruments, after pupil dilation. The same scanning protocol was used for image acquisition. The devices were prototype PLEX Elite 9000 (Carl Zeiss Meditec Inc., Dublin, California, USA) and DRI OCT-A Triton Plus (Topcon Medical Systems Europe, Milano, Italy). Zeiss PLEX Elite uses a 1,060 nm wavelength, with a scanning speed of 100,000 A-scans/second, and image processing is obtained through the so-called OCT-microangiography complex algorithm (OMAG) [23, 24]. Topcon DRI-OCT uses a 1,050 nm wavelength, with a scanning speed of 100,000 A-scans/second and image processing relying on a motion contrast measure named OCT-A Ratio Analysis (OCTARA) [26]. The acquisition protocol performed included the following scans: a linear 12 mm high-definition B-scan centered on the fovea at 0°, OCT-A maps covering the central 3 × 3 mm and 6 × 6 mm macular area. All OCT-A images were carefully reviewed to check automatic segmentations of the superficial capillary plexus (SCP) and deep capillary plexus (DCP), and manual corrections were applied, when necessary, in order to ensure a correct segmentation. For PLEX Elite device, the projections' removal tool was applied for evaluation of DCP. Poor quality images and/or with artifacts were excluded from the analysis.

#### 2.2.2. Quantitative Evaluation of OCT-A Images

Both 3 × 3 mm and 6 × 6 mm OCT-A maps were used for quantitative analysis. All images were saved and analyzed in anonymous and masked fashion. The following quantitative parameters were evaluated: area and circularity index (CI) of the FAZ; perfusion density (PD) and vessel density (VD); and branch analysis including the number of branches (NoB) and total branch length (tBL). All these parameters were evaluated on both SCP and DCP using ImageJ software, version 1.51 (http://imagej.nih.gov/ij/; provided in the public domain by the National Institutes of Health, Bethesda, MD, USA). For DRI-Triton Plus OCT-A, the SCP slab was segmented with an inner boundary at the inner limiting membrane (ILM) +2.6 *μ*m and an outer boundary at the inner plexiform layer (IPL)/inner nuclear layer (INL) +15.6 *μ*m, while the DCP slab was segmented between IPL/INL +15.6 *μ*m and IPL/INL +70.2 *μ*m. For PLEX Elite OCT-A, the SCP slab was segmented between ILM and IPL, while the DCP slab extended from the IPL to the retinal pigment epithelium (RPE fit) −100 *μ*m.

#### 2.2.3. ImageJ Analysis

All DRI-Triton Plus OCT-A images were exported and analyzed with their original resolution of 320 × 320 pixels (9.4 *μ*m lateral resolution for 3 × 3 mm images and 18.7 *μ*m lateral resolution for 6 × 6 mm images). PLEX Elite OCT-A images were exported with their original resolution of 300 × 300 pixels for 3 × 3 mm angiocubes (10 *μ*m lateral resolution) and 500 × 500 pixels for 6 × 6 mm angiocubes (12 *μ*m lateral resolution) and analyzed after a process of cropping in order to match the DRI-Triton Plus's smaller field of view (images were cropped down by about 10%). All images were then opened in ImageJ analysis software.

The FAZ profile was manually outlined using the freehand selection tool on images of SCP and DCP using a previously published method [32], and the software automatically calculated FAZ perimeter and area. FAZ CI was then measured using the following equation: FAZ CI = (4*π* × area)/perimeter^2^. CI is the expression of the regularity of a shape: the more its value is closer to 1, the more the shape is similar to a perfect circle [31].

**Figure 1:**
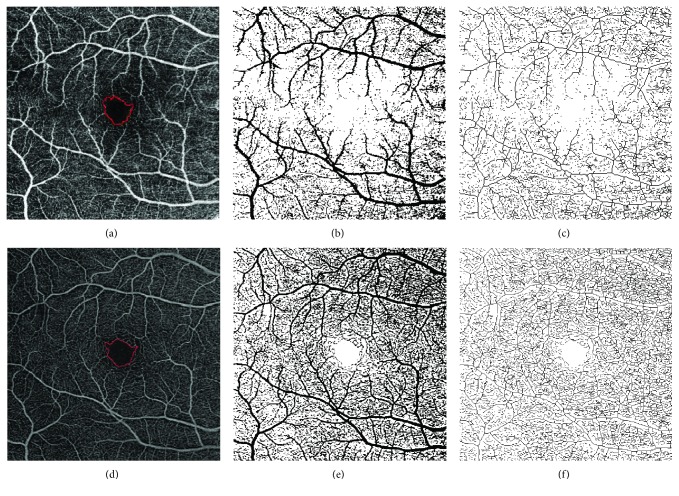
ImageJ analysis of 6 × 6 mm images at the SCP of a patient with DM and without DR. (a–c) SCP image obtained with DRI OCT-A Triton Plus (Topcon Medical Systems Europe, Milano, Italy). (d–f) SCP image obtained with prototype PLEX Elite 9000 (Carl Zeiss Meditec, Inc., Dublin, California, USA). (a, d) Original SCP slabs in which the FAZ profile was manually outlined using the freehand selections tool. (b, e) Binarized images. (c, f) Skeletonized images. SCP: superficial capillary plexus; DM: diabetes mellitus; DR: diabetic retinopathy; OCT-A: OCT angiography; FAZ: foveal avascular zone.

**Figure 2:**
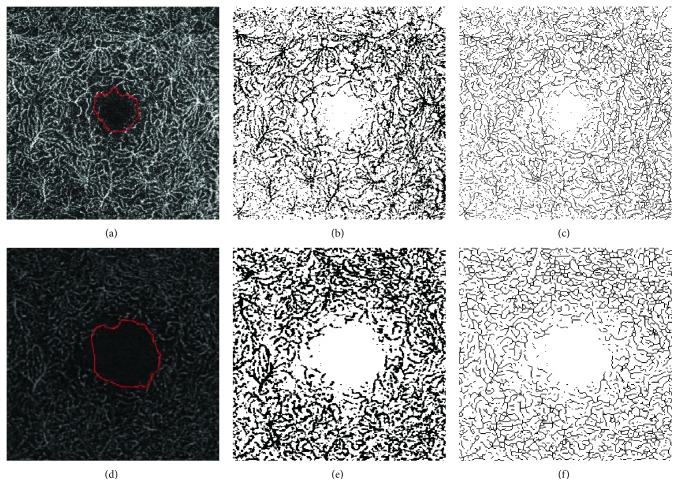
ImageJ analysis of 3 × 3 mm images at the DCP of a patient with DR. (a–c) DCP image obtained with DRI OCT-A Triton Plus (Topcon Medical Systems Europe, Milano, Italy). (d–f) DCP image obtained with prototype PLEX Elite 9000 (Carl Zeiss Meditec, Inc., Dublin, California, USA). (a, d) Original DCP slabs in which the FAZ profile was manually outlined using the freehand selections tool. (b, e) Binarized images. (c, f) Skeletonized images. DCP: deep capillary plexus; DR: diabetic retinopathy; OCT-A: OCT angiography; FAZ: foveal avascular zone.

Images were then converted into 8-bit files, and the Otsu method of thresholding was applied before automatic measurements were performed, as previously reported [33]. Otsu's method of thresholding uses a bimodal distribution and determines the optimum threshold by minimizing intraclass variance and maximizing interclass variance [34]. PD on SCP and DCP (PDS and PDD) was calculated on binarized images as the ratio between all the perfused area in pixels and the total area of the image in pixels. VD on SCP and DCP (VDS and VDD) was calculated after skeletonization of the binarized image; it is a measure of the statistical length of moving the blood column, as previously described [35]. The process of skeletonization reduces all vessel diameter to 1 pixel; therefore, VD has the advantage of not being influenced by vessel dimension (Figures 1 and 2).

The Analyze Skeleton function of ImageJ was then applied to skeletonized images. This plugin tags all pixels in a skeleton image, counts all their junctions, triple and quadruple points, and branches and then measures the average and maximum lengths [36, 37]. When activating this function, a results table called “Branch information” is created; from this table, we considered only two parameters: tBL (total sum of the single branches' length in the area) and NoB (number of branches in the area), as previously described in the peripapillary region of patients with DM [17].

### 2.3. Statistical Analysis

The clinical and demographic variables were compared among the three subject groups using one-way ANOVA. The means of populations were estimated as least square means, which are the best linear estimates for the marginal means in the ANOVA design. In case of an overall statistically significant difference among subject groups, pairwise comparisons among the three groups were done using Scheffé's test.

The ANOVA analyses were performed using statistical version software 6.0 (StatSoft, Inc., Tulsa, OK, USA), using a two-sided type I error rate of *p* ≤ 0.002, after Bonferroni's correction for multiple comparisons.

The agreement between methods was assessed according to the method of Bland-Altman [38]. The mean of the differences (bias), the 95% limits of agreement (LAs), and the 95% confidence intervals for the bias and the LAs were calculated. The distribution of the differences was compared with a Kolmogorov-Smirnoff test to check for normality, as a prerequisite for the Bland-Altman method applicability.

## 3. Results

Of 111 examined subjects/eyes, 73 had no DR (mean age: 51 ± 20.4 years), 20 had mild DR (mean age: 63 ± 14.5 years), and 18 were healthy controls (mean age: 50 ± 21.1 years). There was no significant difference in the mean age among the three groups (one-way ANOVA, *p* = 0.06). Of 93 patients with DM, 38 had type 1 DM and 55 had type 2 DM.

Mean duration of DM was 12.7 ± 10.7 years in the DM with no DR group and 18.3 ± 11.4 in the DR group (*p* = 0.049). Mean value of HbA1c was 7.1 ± 1.1 in the DM with no DR group and 7.6 ± 1.2 in the DR group (*p* = 0.055). Mean BCVA value was 85 ± 0.0 ETDRS letters in the control group, 84.8 ± 1.2 in the DM with no DR group, and 84.3 ± 1.6 in the DR group (*p* = 0.15).

Table 1 shows the mean values of the significant parameters evaluated on 6 × 6 mm angiocubes in different groups. The following parameters were significantly decreased in the DR group versus controls with both instruments: CI and tBL in the SCP and VD and NoB in the DCP. The FAZ area in the DCP was significantly greater with both instruments in the DR group versus the controls. The following parameters were significantly decreased in the DR group versus controls only with PLEX Elite OCT-A: PD and VD in the SCP and PD, FAZ CI, NoB, and tBL in the DCP. The following parameters were significantly different in the no-DR group versus controls: a decrease in PD and tBL in the DCP and an increase in FAZ area in the DCP detected only with PLEX Elite and a decrease in FAZ CI in the SCP detected only with DRI-Triton Plus.

Table 2 shows the mean values of significant parameters evaluated on 3 × 3 mm angiocubes in different groups. The following parameters were significantly decreased in the DR group versus controls: PD, VD, CI, and tBL in the DCP with both instruments; NoB in the DCP with only PLEX Elite; and CI in the SCP with only DRI-Triton Plus. FAZ area in the DCP was significantly greater only with PLEX Elite. FAZ CI in the DCP was significantly reduced only with PLEX Elite in no-DR group versus controls.

**Figure 3:**
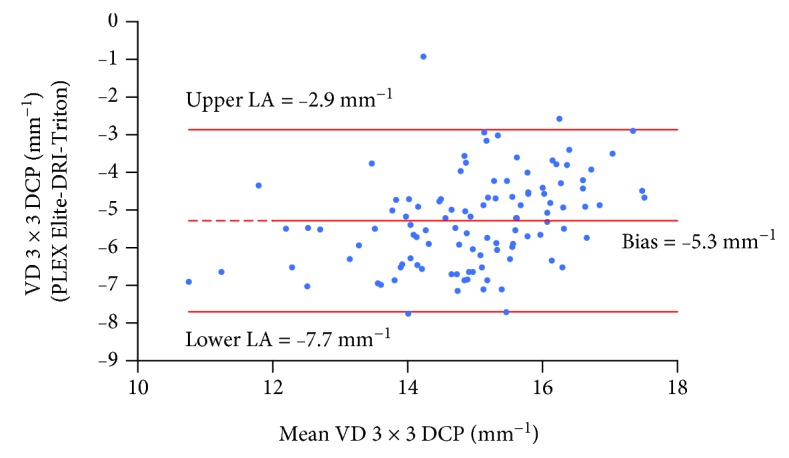
Bland-Altman plot for VD in 3 × 3 mm angiocube scans evaluated at the DCP measured with PLEX Elite and DRI-Triton. The central line indicates the mean of the differences or bias; the upper and lower lines indicate the upper and lower limits of agreement (LA), respectively. VD: vessel density; DCP: deep capillary plexus.

**Figure 4:**
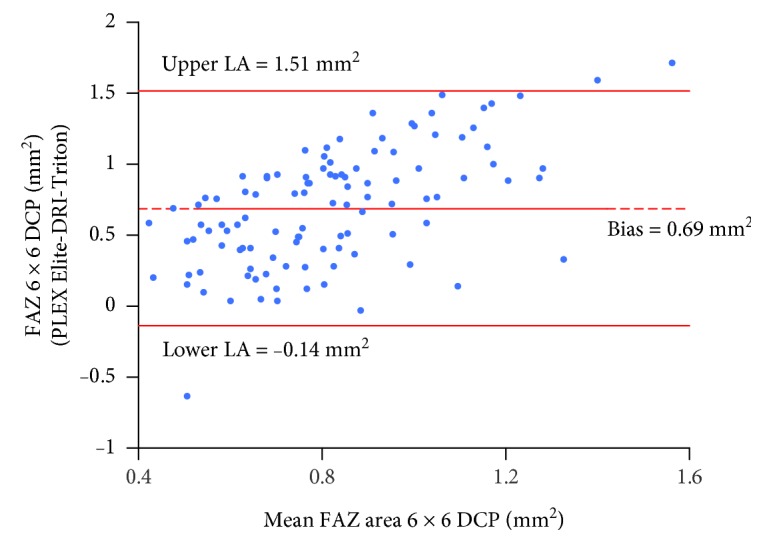
Bland-Altman plot for the FAZ area in 6 × 6 mm angiocube scans evaluated at the DCP measured with PLEX Elite and DRI-Triton. The central line indicates the mean of the differences or bias; the upper and lower lines indicate the upper and lower limits of agreement (LA), respectively. DCP: deep capillary plexus.

Table 3 summarizes the results of Bland-Altman analysis for PD, VD, FAZ, NoB, and tBL, showing comparison between the two OCT-A instruments. A systemic bias exists between the two instruments with PLEX Elite providing higher values for all tested parameters, except for FAZ CI in the SCP, when considering 6 × 6 mm angiocubes. However, when evaluating the 3 × 3 mm angiocube, the difference between the two instruments is less clear, with PLEX Elite providing higher values only for PD and FAZ area. As representative examples, Figure 3 shows the Bland-Altman plot for VD in 3 × 3 mm angiocube scans evaluated at the DCP. The width of the LA's interval is quite narrow, amounting to only 31.5% of the mean value, thus indicating a good agreement between the two instruments.  Figure 4 shows the Bland-Altman plot for the FAZ area in 6 × 6 mm angiocube scans evaluated at the DCP. The width of the LAs' interval is wide, amounting to 197.8% of the mean value, thus indicating a poor agreement between the two instruments.

## 4. Discussion

In the present study, a quantitative evaluation of microvascular changes occurring in the macula in patients with DM with and without clinical signs of DR was performed, using two different SS OCT-A devices and two different angiocube scan sizes. A significant alteration of specific OCT-A parameters was confirmed with both instruments in patients with initial signs of DR when compared to healthy controls.

OCT-A is a method recently introduced in clinical practice that allows for a detailed characterization of retinal microvasculature through the segmentation of individual retinal vascular layers. Recently, Gildea published a review focusing on the diagnostic value of OCT-A in the evaluation of a number of microvascular parameters in patients with diabetes and highlighting the usefulness of this technique in the identification and localization of microaneurysms; visualization of preretinal neovascularization and areas of capillary nonperfusion; detection of FAZ enlargement; and remodeling and quantification of vascular perfusion and branching complexity [39]. However, different OCT-A devices and segmentation methods that have been used as well as different regions of interest have been analyzed in these studies, making it difficult to draw final conclusions, especially when considering quantitative vascular perfusion parameters such as VD and PD [39]. In particular, the majority of available data are obtained with spectral domain OCT-A devices and just few studies were performed with SS-OCT-A. Swept-source OCT-A devices use a longer wavelength (1050 nm), thus having a better ability to penetrate deeper into the tissues than spectral domain devices that use a shorter wavelength. While many studies reported high intra- and interoperator reproducibility in the evaluation of different OCT-A parameters, both in normal and pathologic eyes, using the same scan type and the same device (in particular, FAZ area evaluation at the SCP and perfusion parameters) [40–47], concerns remain on the results interchangeability when using different scan sizes and devices. Rabiolo et al. recently published a study performed with PLEX Elite, comparing FAZ area and VD measurements in different angiocube scan sizes (3 × 3, 6 × 6, and 12 × 12 mm) after cropping original images to obtain the same size. The authors concluded that FAZ area is a robust parameter even if measured on different angiocubes, while VD depends on image size [47].

Different studies performed with OCT-A focused on FAZ measurement as a marker of microvascular damage, documenting that patients with DM had larger FAZ areas versus healthy controls [10, 12–15, 48]. Different methods for quantitative evaluation of FAZ circularity in DM have been recently proposed [49, 50]. In the present study, CI turned out to be an early parameter showing FAZ changes both in the SCP and DCP. Indeed, a clear decreasing trend was documented from controls to no-DR and DR groups, meaning that FAZ regularity was gradually lost as retinal microvascular damage, induced by DM, progressed.

Moreover, the present study documents a significant decrease in VD and PD in patients with initial signs of DR versus healthy controls. This difference was detected with both instruments. These data are in agreement with previously published studies reporting a significant decrease in VD in the macular region in patients with DR compared to healthy controls [51, 52]. In the present study, both angiocube scans (3 × 3 mm and 6 × 6 mm) detected a significant difference in VD and PD evaluated in DCP, while significant differences in the SCP were found only in 6 × 6 mm scans, in particular, using PLEX Elite OCT-A. Hirano et al. evaluated PD and VD on different scan sizes (3 × 3, 6 × 6, and 12 × 12 mm) using PLEX Elite [53]. The results are partially in agreement with our data, reporting a significant decrease in both PD and VD on all scan sizes between healthy controls and eyes with DR, but no significant differences between healthy and diabetic eyes without DR. However, different from what we found, these differences were described both in the DCP and SCP even on 3 × 3 mm images [53].

We would like to point out that two aspects should be considered when discussing the findings reported in the present study. First of all, the two devices use different segmentation methods and have different resolutions. In particular, lateral resolution of the two instruments is similar for the 3 × 3 mm images, while the lateral resolution of PLEX Elite's 6 × 6 mm images is significantly higher compared to that of 6 × 6 mm images acquired with DRI-Triton Plus device. This could explain why PLEX Elite was able to detect significant changes not only in 3 × 3 mm but also in 6 × 6 mm images.

Another important consideration that should be made is that our analysis of 6 × 6 mm images (obtained with PLEX Elite) allowed detecting changes occurring not only in the DCP but also in the SCP. Recent studies performed with OCT-A suggest that changes induced by DM first occur in the DCP and then involve the SCP with disease progression [54–56]. This may be due to a higher density of smaller vessels (more susceptible to hypoxic damage) in the DCP compared to the SCP [57, 58]. Based on our results, we could confirm that lesions induced by diabetes were firstly detectable at the DCP and secondly at the SCP. As the decrease in macular perfusion parameters at SCP level was detected, only on 6 × 6 mm angiocubes and not on 3 × 3 mm angiocubes, we may hypothesize that lesions at the SCP start from a more peripheral macular area and then involve into the inner perifoveal region (more central area). This would need to be confirmed with further studies.

Lastly, in this study, we found a significant reduction in NoB and tBL in patients with DR compared to healthy controls in the macular region. To the best of our knowledge, this is the first study to perform this kind of automatic evaluation of vessel complexity in the macular region. Previously, the same method was used to investigate the peripapillary region of patients with diabetes, finding a significant reduction also in patients with DM without clinical signs of DR when compared to healthy controls [17]. It is hypothesized that NoB and tBL reduction could be a consequence of loss of small branching vessels resulting in reduced branching complexity of retinal vasculature [17, 38]. Previously published studies on OCT-A used a different method, called fractal dimension (FD), to analyze the complexity of retinal microvasculature in the macular region [35, 51, 55, 59–62]. FD was significantly altered in patients with DM when compared to healthy subjects and seemed to be associated with increasing severity of DR [35, 51, 55, 59–62]. Therefore, these studies support the hypothesis that the complexity of microvascular network progressively decreases with increasing severity of DR [35, 51, 55, 59–62].

We performed a Bland-Altman analysis to assess the agreement between the two OCT-A devices used in the present study. We found that the agreement between the two instruments was extremely variable depending on the parameter taken into account. Indeed, LA intervals ranged from acceptable values of ≤50% for some parameters (such as PD and VD) to very high values for some other parameters. In particular, LA intervals > 100% were detected for FAZ area and were probably due to the fact that this was the only parameter evaluated in a noncompletely automatic way (FAZ profile was manually outlined using ImageJ). In addition, the two instruments use different segmentation boundaries to delineate SCP and DCP.

The major limitations of this study include the small sample size of patients with multiple comparisons and the lack of homogeneity in the number of different study groups. However, we decided to use Bonferroni's correction for multiple comparisons in order to reduce the risk of having false-positive results, strengthening the validity of our results. In addition, the power of the study is given by the size of the smallest group (control group); thus, the difference in the group numbers should not influence the final results.

In conclusion, this study documents early microvascular changes occurring in the macular region of patients at the initial stages of DR. These changes were confirmed with both SS OCT-A instruments. Based on these results, we would suggest to perform 3 × 3 mm macular angiocube scans when using DRI-Triton Plus OCT-A, due to its higher resolution. On the other hand, PLEX Elite 6 × 6 mm angiocube scans seem to detect earlier vascular perfusion changes. Therefore, we should be careful in the evaluation of OCT-A results obtained with different devices: the fact that early microvascular alterations could not be seen does not necessarily mean that these alterations are not actually present, but this could be an intrinsic limitation of the device itself. Further, larger longitudinal studies are needed to better understand the exact extent of microvascular damage in very early stages of diabetic retinal disease and to precisely define the strengths and weaknesses of different OCT-A devices and different scan protocols.

## Figures and Tables

**Table 1:** Significant quantitative macular parameters evaluated on 6 × 6 mm angiocubes.

Parameter	Swept-source OCT-A	Controls (*n* = 18)	no-DR (*n* = 73)	DR (*n* = 20)	*p* value^∗^
VDD	DRI-Triton	11.2 ± 0.7	10.9 ± 0.7	10.1 ± 1.0^†^	0.0006
	PLEX Elite	16.2 ± 1.2	14.7 ± 2.8	13.3 ± 1.0^‡^	0.002
FAZ area DCP	DRI-Triton	0.60 ± 0.17	0.42 ± 0.17	0.60 ± 0.25^§^	<0.0001
	PLEX Elite	0.78 ± 0.23	1.22 ± 0.32^¶^	1.37 ± 0.55^¶^	<0.0001
FAZ CI SCP	DRI-Triton	0.90 ± 0.04	0.80 ± 0.109^‡^	0.68 ± 0.19^¶^	<0.0001
	PLEX Elite	0.83 ± 0.07	0.73 ± 0.09	0.64 ± 0.21^¶^	<0.0001
NoB SCP	DRI-Triton	3523 ± 341	3232 ± 488	2801 ± 532	0.002
	PLEX Elite	6691 ± 343	6278 ± 631	5813 ± 794^¶^	0.0002
tBL SCP	DRI-Triton	1.9 × 10^7^ ± 4.5 × 10^6^	2.4 × 10^7^ ± 5.7 × 10^6^	1.6 × 10^7^ ± 3.5 × 10^6^	<0.0001
	PLEX Elite	4.3 × 10^7^ ± 3.5 × 10^6^	3.9 × 10^7^ ± 6.3 × 10^6^	3.5 × 10^7^ ± 7.6 × 10^6^^∆^	0.0004
PDS	PLEX Elite	0.39 ± 0.02	0.37 ± 0.04	0.34 ± 0.05^#^	0.0004
PDD	PLEX Elite	0.44 ± 0.03	0.39 ± 0.03^¶^	0.36 ± 0.03^¶,∆^	<0.0001
VDS	PLEX Elite	13.2 ± 0.8	12.4 ± 1.4	11.4 ± 1.8^†^	0.001
FAZ CI DCP	PLEX Elite	0.83 ± 0.06	0.76 ± 0.10	0.69 ± 0.10^¶^	<0.0001
NoB DCP	PLEX Elite	8064 ± 944	7621 ± 413	7128 ± 469^¶,‡^	<0.0001
tBL DCP	PLEX Elite	5.8 × 10^7^ ± 8.5 × 10^6^	4.9 × 10^7^ ± 5.3 × 10^6^^¶^	4.3 × 10^7^ ± 4.3 × 10^6^^¶,||^	<0.0001

^∗^One-way ANOVA analyses: comparison among controls, patients with DM without DR, and patients with DR. Statistical significance was set at *p* = 0.002 after Bonferroni's correction. Comparison versus controls: ^†^Scheffé's test, *p* = 0.001; ^‡^Scheffé's test, *p* = 0.002; ^¶^Scheffé's test, *p* < 0.0001; ^#^Scheffé's test, *p* = 0.0004. Comparison versus patients with DM without DR: ^§^Scheffé's test, *p* = 0.002; ^∫^Scheffé's test, *p* = 0.0002; ^∆^Scheffé's test, *p* < 0.0001; ^||^Scheffé's test, *p* = 0.0003. VDD: vessel density at the deep capillary plexus; FAZ: foveal avascular zone; DCP: deep capillary plexus; CI: circularity index; SCP: superficial capillary plexus; NoB: number of branches; tBL: total branches length; PDS: perfusion density at the superficial capillary plexus; PDD: perfusion density at the deep capillary plexus; VDS: vessel density at the superficial capillary plexus.

**Table 2:** Significant quantitative macular parameters evaluated on 3 × 3 mm angiocubes.

Parameter	Swept-source OCT-A	Controls (*n* = 18)	no-DR (*n* = 73)	DR (*n* = 20)	*p* value^∗^
PDD	DRI-Triton	0.31 ± 0.02	0.32 ± 0.02	0.30 ± 0.03^§^	0.0001
	PLEX Elite	0.37 ± 0.05	0.36 ± 0.04	0.31 ± 0.05^#,‡^	0.0001
VDD	DRI-Triton	18.0 ± 1.3	18.2 ± 1.2	16.8 ± 1.4^∫^	0.0001
	PLEX Elite	13.5 ± 1.9	12.8 ± 1.4	11.2 ± 1.8^†,||^	0.0001
FAZ CI DCP	DRI-Triton	0.75 ± 0.08	0.75 ± 0.09	0.65±0.19^∗∗^	0.001
	PLEX Elite	0.82 ± 0.05	0.67 ± 0.13^¶^	0.64 ± 0.11^¶^	<0.0001
tBL DCP	DRI-Triton	2.1 × 10^7^ ± 1.9 × 10^6^	2.2 × 10^7^ ± 2.1 × 10^6^	2.0 × 10^7^ ± 2.4 × 10^6^^∫∫^	0.0007
	PLEX Elite	1.3 × 10^7^ ± 2.3 × 10^6^	1.2 × 10^7^ ± 1.7 × 10^6^	1.1 × 10^7^ ± 1.8 × 10^6^^‡‡^	0.0007
FAZ CI SCP	DRI-Triton	0.72 ± 0.09	0.73 ± 0.12	0.57±0.20^∗∗^^,∆^	0.0002
FAZ area DCP	PLEX Elite	1.06 ± 0.25	1.37 ± 0.32	1.54 ± 0.54^#^	0.0003
NoB DCP	PLEX Elite	1861 ± 282	1765 ± 220	1551 ± 216^¶¶,§^	0.0002

^∗^One-way ANOVA analyses: comparison among controls, patients with DM without DR and patients with DR. Statistical significance was set at *p* = 0.002 after Bonferroni's correction. Comparison versus controls: ^#^Scheffé's test, *p* = 0.0003; ^†^Scheffé's test, *p* = 0.0001; ^∗∗^Scheffé's test, *p* = 0.002; ^¶^Scheffé's test, *p* < 0.0001; ^‡‡^Scheffé's test, *p* = 0.001; ^¶¶^Scheffé's test, *p* = 0.0004. Comparison versus patients with DM without DR: ^§^Scheffé's test, *p* = 0.002; ^‡^Scheffé's test, *p* = 0.0006; ^∫^Scheffé's test, *p* = 0.0001; ^||^Scheffé's test, *p* = 0.001; ^∫∫^Scheffé's test, *p* = 0.0009; ^∆^Scheffé's test, *p* < 0.0001. PDD: perfusion density at the deep capillary plexus; VDD: vessel density at the deep capillary plexus; FAZ: foveal avascular zone; CI: circularity index; DCP: deep capillary plexus; tBL: total branches length; SCP: superficial capillary plexus; NoB: number of branches.

**Table 3:** Results of Bland-Altman analysis for PD, VD, FAZ, NoB, and tBL for comparisons between methods A (DRI-Triton) and B (PLEX Elite).

Parameter	Bias^∗^ [95% CI]	Lower LA [95% CI]	Upper LA [95% CI]	LA interval (%)^∗∗^
*PD*				
3 × 3 SCP	0.091 [0.083, 0.098]	0.01 [0.00, 0.03]	0.17 [0.15, 0.20]	47.8
3 × 3 DCP	0.033 [0.026, 0.041]	-0.04 [-0.06, -0.03]	0.11 [0.10, 0.12]	44.8
6 × 6 SCP	0.11 [0.097, 0.114]	0.02 [0.004, 0,03]	0.19 [0.18, 0.21]	53.9
6 × 6 DCP	0.087 [0.080, 0.094]	0.01 [0.00, 0.03]	0.16 [0.15, 0.20]	42.3
*VD*				
3 × 3 SCP	-0.6 [-0.95, -0.25]	-4.2 [-4.8, -3.6]	3.0 [2.4, 3.6]	51.5
3 × 3 DCP	-5.3 [-5.5, -5.1]	-7.7 [-8.1, -7.3]	-2.9 [-3.3, -2.5]	31.5
6 × 6 SCP	4.5 [4.3,4.7]	2.3 [2.0, 2.7]	6.6 [6.3, 7.0]	42.7
6 × 6 DCP	3,8 [3.6,4.0]	1.5 [1.1, 1.9]	6.1 [5.7, 6.4]	36.1
*FAZ*				
Area 3 × 3 SCP	0.03 [0.00, 0.6]	-0.28 [-0.33, -0.22]	0.33 [0.28, 0.38]	191.1
Area 3 × 3 DCP	0.85 [0.78, 0.91]	0.20 [0.09, 0.31]	1.49 [1.38, 1.60]	138.9
Area 6 × 6 SCP	0.01 [-0.01, 0.03]	-0.19 [-0.22, -0.16]	0.020 [0.17, 0.24]	125.2
Area 6 × 6 DCP	0.69 [0.61, 0.77]	-0.14 [-0.28, 0.00]	1.51 [1.37, 1.65]	197.8
CI 3 × 3 SCP	-0.05 [-0.07, -0.02]	-0.31 [-0.36, -0.27]	0.22 [0.17, 0.26]	76.7
CI 3 × 3 DCP	-0.06 [-0.09, -0.03]	-0.36 [-0.41, -0.31]	0.24 [0.19, 0.30]	83.8
CI 6 × 6 SCP	-0.06 [-0.08, -0.05]	-0.27 [-0.30, -0.23]	0.14 [0.10, 0.17]	51.8
CI 6 × 6 DCP	0.04 [0.00, 0.07]	-0.29 [-0.34, -0.23]	0.36 [0.30, 0.41]	87.2
*NoB*				
3x3 SCP	-732 [-795, -669]	-1378 [-1487, -1269]	-85 [-194, 24]	57.2
3 × 3 DCP	-1681 [-1713, -1648]	-2015 [-2072, -1959]	-1346 [-1403, -1290]	25.9
6 × 6 SCP	3104 [3006, 3202]	2098 [1929, 2268]	4110 [3940, 4279]	42.7
6 × 6 DCP	3029 [2902, 3157]	1717 [1496, 1938]	0.36 [4120, 4562]	43.0
*tBL*				
3 × 3 SCP	-2.9 × 10^6^ [-3.4 × 10^6^, -2.4 × 10^6^]	-7.7 × 10^6^ [-8.5 × 10^6^, -6.9 × 10^6^]	1.9 × 10^6^ [1.1 × 10^6^, 2.7 × 10^6^]	68.7
3 × 3 DCP	-9.5 × 10^6^ [−9.9 × 10^6^, -9.1 × 10^6^]	−13.6 × 10^6^ [-14.3 × 10^6^, -12.9 × 10^6^]	−5.4 × 10^6^ [-6.1 × 10^6^, -4.7 × 10^6^]	49.3
6 × 6 SCP	17.5 × 10^6^ [15.9 × 10^6^, 19.1 × 10^6^]	1.1 × 10^6^ [-1.6 × 10^6^, 3.9 × 10^6^]	33.9 × 10^6^ [31.1 × 10^6^, 36.6 × 10^6^]	107.3
6 × 6 DCP	16.1 × 10^6^ [14.3 × 10^6^, 17.8 × 10^6^]	-2.3 × 10^6^ [-5.4 × 10^6^, 0.8 × 10^6^]	33.9 × 10^6^ [31.3 × 10^6^, 37.5 × 10^6^]	88.3

^∗^Comparisons were always performed considering the difference between method B (PLEX Elite) and method A (DRI-Triton). Thus, a positive bias means PLEX Elite mean values are greater than those of DRI-Triton's. ^∗∗^LA interval was calculated and the ratio between the amplitude of the interval (difference between upper LA and lower LA) and the mean value of the considered parameter in percentage. PD: perfusion density; SCP: superficial capillary plexus; DCP: deep capillary plexus; VD: vessel density; FAZ: foveal avascular zone; CI: circularity index; NoB: number of branches; tBL: total branches length; LA: limits of agreement.

## Data Availability

The data used to support the findings of this study are included within the article.

## References

[1] Yau J. W. Y., Rogers S. L., Kawasaki R. (2012). Global prevalence and major risk factors of diabetic retinopathy. *Diabetes Care*.

[2] Gardner T. W., Abcouwer S. F., Barber A. J., Jackson G. R. (2011). An integrated approach to diabetic retinopathy research. *Archives of Ophthalmology*.

[3] Joussen A. M., Poulaki V., Qin W. (2002). Retinal vascular endothelial growth factor induces intercellular adhesion molecule-1 and endothelial nitric oxide synthase expression and initiates early diabetic retinal leukocyte adhesion *in vivo*. *The American Journal of Pathology*.

[4] Martin P. M., Roon P., van Ells T. K., Ganapathy V., Smith S. B. (2004). Death of retinal neurons in streptozotocin-induced diabetic mice. *Investigative Opthalmology & Visual Science*.

[5] Lecleire-Collet A., Tessier L. H., Massin P. (2005). Advanced glycation end products can induce glial reaction and neuronal degeneration in retinal explants. *British Journal of Ophthalmology*.

[6] Antonetti D. A., Barber A. J., Bronson S. K. (2006). Diabetic retinopathy: seeing beyond glucose-induced microvascular disease. *Diabetes*.

[7] Lung J. C. Y., Swann P. G., Wong D. S. H., Chan H. H. L. (2012). Global flash multifocal electroretinogram: early detection of local functional changes and its correlations with optical coherence tomography and visual field tests in diabetic eyes. *Documenta Ophthalmologica*.

[8] Vujosevic S., Micera A., Bini S., Berton M., Esposito G., Midena E. (2016). Proteome analysis of retinal glia cells-related inflammatory cytokines in the aqueous humour of diabetic patients. *Acta Ophthalmologica*.

[9] Vujosevic S., Micera A., Bini S., Berton M., Esposito G., Midena E. (2015). Aqueous humor biomarkers of Müller cell activation in diabetic eyes. *Investigative Opthalmology & Visual Science*.

[10] Vujosevic S., Muraca A., Alkabes M. (2019). Early microvascular and neural changes in patients with type 1 and type 2 diabetes mellitus without clinical signs of diabetic retinopathy. *Retina*.

[11] Sohn E. H., van Dijk H. W., Jiao C. (2016). Retinal neurodegeneration may precede microvascular changes characteristic of diabetic retinopathy in diabetes mellitus. *Proceedings of the National Academy of Sciences of the United States of America*.

[12] de Carlo T. E., Chin A. T., Bonini Filho M. A. (2015). Detection of microvascular changes in eyes of patients with diabetes but not clinical diabetic retinopathy using optical coherence tomography angiography. *Retina*.

[13] Dimitrova G., Chihara E., Takahashi H., Amano H., Okazaki K. (2017). Quantitative retinal optical coherence tomography angiography in patients with diabetes without diabetic retinopathy. *Investigative Opthalmology & Visual Science*.

[14] Di G., Weihong Y., Xiao Z. (2016). A morphological study of the foveal avascular zone in patients with diabetes mellitus using optical coherence tomography angiography. *Graefe's Archive for Clinical and Experimental Ophthalmology*.

[15] Takase N., Nozaki M., Kato A., Ozeki H., Yoshida M., Ogura Y. (2015). Enlargement of foveal avascular zone in diabetic eyes evaluated by en face optical coherence tomography angiography. *Retina*.

[16] Cao D., Yang D., Huang Z. (2018). Optical coherence tomography angiography discerns preclinical diabetic retinopathy in eyes of patients with type 2 diabetes without clinical diabetic retinopathy. *Acta Diabetologica*.

[17] Vujosevic S., Muraca A., Gatti V. (2018). Peripapillary microvascular and neural changes in diabetes mellitus: an OCT-angiography study. *Investigative Opthalmology & Visual Science*.

[18] Lee D.-H., Yi H. C., Bae S. H., Cho J. H., Choi S. W., Kim H. (2018). Risk factors for retinal microvascular impairment in type 2 diabetic patients without diabetic retinopathy. *PLoS One*.

[19] Kim K., Kim E. S., Yu S. Y. (2018). Optical coherence tomography angiography analysis of foveal microvascular changes and inner retinal layer thinning in patients with diabetes. *British Journal of Ophthalmology*.

[20] Scarinci F., Picconi F., Virgili G. (2017). Single retinal layer evaluation in patients with type 1 diabetes with no or early signs of diabetic retinopathy: the first hint of neurovascular crosstalk damage between neurons and capillaries?. *Ophthalmologica*.

[21] Simonett J. M., Scarinci F., Picconi F. (2017). Early microvascular retinal changes in optical coherence tomography angiography in patients with type 1 diabetes mellitus. *Acta Ophthalmologica*.

[22] Rabiolo A., Carnevali A., Bandello F., Querques G. (2016). Optical coherence tomography angiography: evolution or revolution?. *Expert Review of Ophthalmology*.

[23] Rosenfeld P. J., Durbin M. K., Roisman L. (2016). ZEISS Angioplex^TM^ spectral domain optical coherence tomography angiography: technical aspects. *Developments in Ophthalmology*.

[24] Miller A. R., Roisman L., Zhang Q. (2017). Comparison between spectral-domain and swept-source optical coherence tomography angiographic imaging of choroidal neovascularization. *Investigative Opthalmology & Visual Science*.

[25] Huang D., Jia Y., Gao S. S., Lumbroso B., Rispoli M. (2016). Optical coherence tomography angiography using the Optovue device. *Developments in Ophthalmology*.

[26] Huang Y., Zhang Q., Thorell M. R. (2014). Swept-source OCT angiography of the retinal vasculature using intensity differentiation-based optical microangiography algorithms. *Ophthalmic Surgery, Lasers and Imaging Retina*.

[27] Coscas G., Lupidi M., Coscas F. (2016). Heidelberg spectralis optical coherence tomography angiography: technical aspects. *Developments in Ophthalmology*.

[28] Corvi F., Pellegrini M., Erba S., Cozzi M., Staurenghi G., Giani A. (2018). Reproducibility of vessel density, fractal dimension, and foveal avascular zone using 7 different optical coherence tomography angiography devices. *American Journal of Ophthalmology*.

[29] American Diabetes Association (2018). 2. Classification and diagnosis of diabetes: standards of medical care in diabetes—2019. *Diabetes Care*.

[30] Wilkinson C. P., Ferris F. L., Klein R. E. (2003). Proposed international clinical diabetic retinopathy and diabetic macular edema disease severity scales. *Ophthalmology*.

[31] Mancia G., Fagard R., Narkiewicz K. (2013). 2013 ESH/ESC guidelines for the management of arterial hypertension: the Task Force for the management of arterial hypertension of the European Society of Hypertension (ESH) and of the European Society of Cardiology (ESC). *European Heart Journal*.

[32] Choi J., Kwon J., Shin J. W., Lee J., Lee S., Kook M. S. (2017). Quantitative optical coherence tomography angiography of macular vascular structure and foveal avascular zone in glaucoma. *PloS One*.

[33] Al-Sheikh M., Phasukkijwatana N., Dolz-Marco R. (2017). Quantitative OCT angiography of the retinal microvasculature and the choriocapillaris in myopic eyes. *Investigative Opthalmology & Visual Science*.

[34] Schneider C. A., Rasband W. S., Eliceiri K. W. (2012). NIH Image to ImageJ: 25 years of image analysis. *Nature Methods*.

[35] Reif R., Qin J., An L., Zhi Z., Dziennis S., Wang R. (2012). Quantifying optical microangiography images obtained from a spectral domain optical coherence tomography system. *International Journal of Biomedical Imaging*.

[36] Arganda-Carreras I., Fernández-González R., Muñoz-Barrutia A., Ortiz-De-Solorzano C. (2010). 3D reconstruction of histological sections: application to mammary gland tissue. *Microscopy Research and Technique*.

[37] Polder G., Hovens H. L. E., Zweers A. J., Jahnen A., Moll C., Kennedy A. J. F. Measuring shoot length of submerged aquatic plants using graph analysis.

[38] Bland J. M., Altman D. G. (1986). Statistical methods for assessing agreement between two methods of clinical measurement. *The Lancet*.

[39] Gildea D. (2018). The diagnostic value of optical coherence tomography angiography in diabetic retinopathy: a systematic review. *International Ophthalmology*.

[40] Al-Sheikh M., Tepelus T. C., Nazikyan T., Sadda S. V. R. (2017). Repeatability of automated vessel density measurements using optical coherence tomography angiography. *British Journal of Ophthalmology*.

[41] Carpineto P., Mastropasqua R., Marchini G., Toto L., Di Nicola M., Di Antonio L. (2016). Reproducibility and repeatability of foveal avascular zone measurements in healthy subjects by optical coherence tomography angiography. *British Journal of Ophthalmology*.

[42] Guo J., She X., Liu X., Sun X. (2017). Repeatability and reproducibility of foveal avascular zone area measurements using AngioPlex spectral domain optical coherence tomography angiography in healthy subjects. *Ophthalmologica*.

[43] La Spina C., Carnevali A., Marchese A., Querques G., Bandello F. (2017). Reproducibility and reliability of optical coherence tomography angiography for foveal avascular zone evaluation and measurement in different settings. *Retina*.

[44] Mastropasqua R., Toto L., Mattei P. A. (2017). Reproducibility and repeatability of foveal avascular zone area measurements using swept-source optical coherence tomography angiography in healthy subjects. *European Journal of Ophthalmology*.

[45] Shahlaee A., Pefkianaki M., Hsu J., Ho A. C. (2016). Measurement of foveal avascular zone dimensions and its reliability in healthy eyes using optical coherence tomography angiography. *American Journal of Ophthalmology*.

[46] You Q., Freeman W. R., Weinreb R. N. (2017). Reproducibility of vessel density measurement with optical coherence tomography angiography in eyes with and without retinopathy. *Retina*.

[47] Rabiolo A., Gelormini F., Marchese A. (2018). Macular perfusion parameters in different angiocube sizes: does the size matter in quantitative optical coherence tomography angiography?. *Investigative Opthalmology & Visual Science*.

[48] Al-Sheikh M., Akil H., Pfau M., Sadda S. V. R. (2016). Swept-source OCT angiography imaging of the foveal avascular zone and macular capillary network density in diabetic retinopathy. *Investigative Opthalmology & Visual Science*.

[49] Alam M., Zhang Y., Lim J. I., Chan R. V. P., Yang M., Yao X. (2018). Quantitative optical coherence tomography angiography features for objective classification and staging of diabetic retinopathy. *Retina*.

[50] Krawitz B. D., Mo S., Geyman L. S. (2017). Acircularity index and axis ratio of the foveal avascular zone in diabetic eyes and healthy controls measured by optical coherence tomography angiography. *Vision Research*.

[51] Kim A. Y., Chu Z., Shahidzadeh A., Wang R. K., Puliafito C. A., Kashani A. H. (2016). Quantifying microvascular density and morphology in diabetic retinopathy using spectral-domain optical coherence tomography angiography. *Investigative Opthalmology & Visual Science*.

[52] Agemy S. A., Scripsema N. K., Shah C. M. (2015). Retinal vascular perfusion density mapping using optical coherence tomography angiography in normals and diabetic retinopathy patients. *Retina*.

[53] Hirano T., Kitahara J., Toriyama Y., Kasamatsu H., Murata T., Sadda S. (2019). Quantifying vascular density and morphology using different swept-source optical coherence tomography angiographic scan patterns in diabetic retinopathy. *British Journal of Ophthalmology*.

[54] Kim M., Choi S. Y., Park Y. H. (2018). Quantitative analysis of retinal and choroidal microvascular changes in patients with diabetes. *Scientific Reports*.

[55] Chen Q., Ma Q., Wu C. (2017). Macular vascular fractal dimension in the deep capillary layer as an early indicator of microvascular loss for retinopathy in type 2 diabetic patients. *Investigative Opthalmology & Visual Science*.

[56] Ashraf M., Nesper P. L., Jampol L. M., Yu F., Fawzi A. A. (2018). Statistical model of optical coherence tomography angiography parameters that correlate with severity of diabetic retinopathy. *Investigative Opthalmology & Visual Science*.

[57] Savastano M. C., Lumbroso B., Rispoli M. (2015). In vivo characterization of retinal vascularization morphology using optical coherence tomography angiography. *Retina*.

[58] McLenachan S., Magno A. L., Ramos D. (2015). Angiography reveals novel features of the retinal vasculature in healthy and diabetic mice. *Experimental Eye Research*.

[59] Zahid S., Dolz-Marco R., Freund K. B. (2016). Fractal dimensional analysis of optical coherence tomography angiography in eyes with diabetic retinopathy. *Investigative Opthalmology & Visual Science*.

[60] Tang F. Y., Ng D. S., Lam A. (2017). Determinants of quantitative optical coherence tomography angiography metrics in patients with diabetes. *Scientific Reports*.

[61] Bhardwaj S., Tsui E., Zahid S. (2018). Value of fractal analysis of optical coherence tomography angiography in various stages of diabetic retinopathy. *Retina*.

[62] Zhu T., Ma J., Li J. (2019). Multifractal and lacunarity analyses of microvascular morphology in eyes with diabetic retinopathy: a projection artifact resolved optical coherence tomography angiography study. *Microcirculation*.

